# The worldwide spread of *Aedes albopictus*: New insights from mitogenomes

**DOI:** 10.3389/fgene.2022.931163

**Published:** 2022-08-26

**Authors:** Vincenza Battaglia, Vincenzo Agostini, Elisabetta Moroni, Giulia Colombo, Gianluca Lombardo, Nicola Rambaldi Migliore, Paolo Gabrieli, Maria Garofalo, Stella Gagliardi, Ludvik M. Gomulski, Luca Ferretti, Ornella Semino, Anna R. Malacrida, Giuliano Gasperi, Alessandro Achilli, Antonio Torroni, Anna Olivieri

**Affiliations:** ^1^ Dipartimento di Biologia e Biotecnologie “L. Spallanzani”, Università di Pavia, Pavia, Italy; ^2^ Department of Biosciences and Pediatric Clinical Research Center “Romeo ed Enrica Invernizzi”, University of Milan, Milan, Italy; ^3^ Molecular Biology and Transcriptomic Unit, IRCCS Mondino Foundation, Pavia, Italy

**Keywords:** *Aedes albopictus* spread, MtDNA variation, haplogroups, sources of adventive populations, mitogenome, phylogeny

## Abstract

The tiger mosquito (*Aedes albopictus)* is one of the most invasive species in the world and a competent vector for numerous arboviruses, thus the study and monitoring of its fast worldwide spread is crucial for global public health. The small extra-nuclear and maternally-inherited mitochondrial DNA represents a key tool for reconstructing phylogenetic and phylogeographic relationships within a species, especially when analyzed at the mitogenome level. Here the mitogenome variation of 76 tiger mosquitoes, 37 of which new and collected from both wild adventive populations and laboratory strains, was investigated. This analysis significantly improved the global mtDNA phylogeny of *Ae. albopictus*, uncovering new branches and sub-branches within haplogroup A1, the one involved in its recent worldwide spread. Our phylogeographic approach shows that the current distribution of tiger mosquito mitogenome variation has been strongly affected by clonal and sub-clonal founder events, sometimes involving wide geographic areas, even across continents, thus shedding light on the Asian sources of worldwide adventive populations. In particular, different starting points for the two major clades within A1 are suggested, with A1a spreading mainly along temperate areas from Japanese and Chinese sources, and A1b arising and mainly diffusing in tropical areas from a South Asian source.

## Introduction

Native to the tropical forests of Southeast Asia (Gratz, 2004), the tiger mosquito *Aedes (Stegomyia) albopictus* (Skuse, 1894) has colonized every continent except Antarctica over the last 50 years (Benedict et al., 2007; Medlock et al., 2015), and is one of the 100 most invasive species in the world, according to the Global Invasive Species Database (Global Invasive Species Database, 2010).

The initial spread began with the southeastern Asian zoophilic forest species’ move westwards to the Indian Ocean islands and eastwards to the Pacific Ocean Islands in the 19th century and in the first half of the 20th century, respectively (Knudsen, 1995). However, the species remained confined to those areas for the subsequent decades and its worldwide diffusion only started in the early 1980’s (Benedict et al., 2007; Medlock et al., 2015). Since then, *Ae. albopictus* has become established in the Mediterranean basin; the Americas, ranging from the United States to Argentina; Africa, where it first colonized South Africa and is now also present in the central and northern regions of the continent as well; the Middle East; Australia and the rest of Europe. In Europe, after its first occurrence in Albania in 1979 (Adhami and Murati, 1987; Benedict et al., 2007), the tiger mosquito started to expand across the continent from the 90s onward, when it was introduced into Italy through the port of Genoa (Sabatini et al., 1990; Scholte and Schaffner, 2007). Currently, it is established, or at least introduced, in most western and central European countries (European Center for Disease Prevention and Control, 2022).

This rapid and widespread expansion is due to the strong adaptability of the tiger mosquito to lay eggs in anthropized habitats, its diurnal biting habits and its preferential feeding on human blood (Benelli et al., 2020). Major roles are also played by the diapause as main strategy in enhancing overwinter survival, by desiccation-resistant eggs (Pasquali et al., 2020; Vega-Rùa et al., 2020), in addition to globalization, which favored rather frequent human-aided accidental transfer (mediated for example by commerce of plants and used vehicle tyres) (Ibáñez-Justicia et al., 2020), and a diffused lack of efficient surveillance and control methods. Moreover, climatic conditions, such as temperature and precipitations, strongly impact on its diffusion, and climate change is accelerating its spread towards the warming northern regions (Neteler et al., 2011; Roiz et al., 2011; Roiz et al., 2014). In Europe, recent climate changes have pushed *Ae. albopictus* to Moldova, where it was first detected in 2020 (Sulesco et al., 2021), with further introduction events been reported north of the currently colonized area, i.e. in Austria, Switzerland, and Belgium (Schoener et al., 2019).

Understanding and monitoring the tiger mosquito’s worldwide spread is of crucial importance not only due to its ecological and social impact, but also for global public health, since *Ae. albopictus* is a key competent vector for numerous arboviruses, like the yellow fever virus, dengue viruses, chikungunya virus, West Nile virus, Zika virus and Japanese encephalitis virus (Kraemer et al., 2015; Marconcini et al., 2021), and its recent expansion has caused severe outbreaks in many urban populations (Manni et al., 2017; Kraemer et al., 2019; Mariconti et al., 2019; Vega-Rùa et al., 2021).

Population genetics tools give access to a wealth of information on demography, geographic movements and links among populations, and the study of mitochondrial DNA (mtDNA) is best able to quickly reconstruct phylogenetic and phylogeographic relationships (Torroni et al., 2006; Gao et al., 2021). MtDNA is generally about 17 kb long in animals, thus given its size it may appear irrelevant compared to its nuclear counterpart. However, in contrast to autosomal markers, mtDNA is inherited along strict maternal lines of descent and its relatively fast molecular differentiation occurs only through the sequential accumulation of novel mutations, which often takes place during and after populations or species spread into different regions (Torroni et al., 2006; Goubert et al., 2016; da Silva et al., 2020). Thus, novel haplotypes or groups of related haplotypes (haplogroups) tend to be restricted to specific geographic areas and populations. MtDNA studies in (non-human) animals have now entered a new phase: from the analysis of short segments (generally 1 kb or less in length, either a portion of the control-region or of single protein-coding genes such as *COI*, *ND2*, *ND5*, or *CYB*) to the mitogenome-level analyses of a number of individuals (population mitogenomics) (Ruiling et al., 2018a; Fang et al., 2018; Lee et al., 2020). The mitogenome approach can be applied to all animal species, including wild invasive species such as mosquitoes. In 2016, Battaglia and co-workers (Battaglia et al., 2016) published the first entire mitogenome from a mosquito of the Italian (Rimini) laboratory-maintained strain, for which the nuclear genome is also available (Dritsou et al., 2015). This sequence, employed as reference sequence (GenBank accession number KX383916), was used to number and annotate the *Ae. albopictus* mitogenome. To identify the ancestral source(s) of *Ae. albopictus* adventive populations, Battaglia and co-workers analyzed the sequence variation at the level of the entire coding region of 27 mitogenomes, by using a phylogeographic approach. The analysis revealed that the mitogenomes clustered into five major haplogroups, namely A1a1, A1a2, A1b, A2, and A3. Among these, only the three A1 subclades were involved in the recent worldwide spread of the species, with the A1a1a1 sub-clade possibly having arisen in North America from a Japanese source. As for the other two clades, haplogroup A2 encompasses only samples from the Philippines, insular South-East Asia and the North Australian border area (Beebe et al., 2013). In light of this geographical distribution, it has been proposed that haplogroup A2 played a role in the human-mediated spread of *Ae. albopictus* from South-East Asia (in particular from Indonesia), towards northern Oceania (Žitko et al., 2011; Beebe et al., 2013; Zhong et al., 2013; Ismail et al., 2017; Tedjou et al., 2019). Whereas, haplogroup A3 includes only a single mitogenome from Taiwan, which suggests it might be rare and/or restricted to a specific geographic area, and that mosquitoes within this haplogroup are unlikely to be involved in the recent worldwide spread of the species.

Battaglia and co-workers’ study (2016) remained for years the only contribution to the analysis of mtDNA variability in the tiger mosquito at the mitogenome-level, while other studies continued to limit their survey to partial mtDNA sequences (Žitko et al., 2011; Beebe et al., 2013; Zhong et al., 2013; Ismail et al., 2017; Tedjou et al., 2019). In 2017, however, 17 novel mitogenomes from Algarve and Oporto, the two entry sites of the tiger mosquito into Portugal, were published, together with 31 *COI* sequences (Zé-Zé et al., 2020). With this addition, the mitogenome phylogeny improved considerably, but the novel sequences were from only two locations, thus with limited phylogeographic impact.

The present study analyzed, in the framework of available data, the mitogenome variation of 37 new specimens, 29 of which came from wild populations, mainly collected in countries or regions that were not included in previous studies. Our aim was to improve the mtDNA phylogeny of *Ae. albopictus* and better assess the geographic distributions of the previously identified haplogroups, with a particular focus on haplogroup A1, the one involved in the recent worldwide spread.

## Materials and methods

### Sample

A total of 37 novel mosquito samples were included in this study. Of these, 29 were from wild populations collected in Africa, the Americas, Asia and Europe (Table 1). In particular, four were from Cameroon, in Africa; 15 from the Americas: four from Florida (Vero Beach), six from California (Los Angeles), one from Mexico (Tapachula) and four from Brazil (Rio de Janeiro); four from Japan: two from Wakayama, one from Fukushima, and one from Tokyo; six from Europe: two from Italy (one from Reggio Emilia and one from Perugia) and four from France (all from Cuers, Var Department). The remaining eight were adults from laboratory-maintained strains: one from the Japanese Ikuta strain (Kanagawa Prefecture), three from the Chinese Foshan strain (Center for Disease Control and Prevention of Guangdong Province), three from the Foshan inbred strain Pavia A (FPA) and one from the Crema strain (Laboratory of Genomics and Biotechnology of Insects of Agricultural and Medical Importance, University of Pavia) (Palatini et al., 2020). These novel mitogenomes were analyzed together with those previously published (Battaglia et al., 2016; Zé-Zé et al., 2020) (Table 1). The study did not involve protected species and samples were not collected at protected sites.

**TABLE 1:** Origin and haplogroup affiliation of *Ae. albopictus* mitogenomes analysed in this study. The 37 newly sequenced samples are included together with 39 from previous studies. Haplogroups indicated in bold in the Table are new or re-defined relative to (Battaglia et al., 2016).

Sequence ID#^a^	Original name	Continent	Country (place of collection)	Haplogroup	GenBank ID	Number of type II repeats^b^	References
1	Rim1^c^	Europe	Italy, Rimini	**A1a1a1a1**	KX383916	5	Battaglia et al. (2016)
2	J-To1	Asia	Japan, Tokyo	**A1a1a1a1**	MH587188	5	This study
3	J-Ka1^c^	Asia	Japan, Kanagawa	**A1a1a1a1**	MH587189	5	This study
4	VB2	America	United States, Florida, Vero Beach	**A1a1a1a1**	MH587190	5	This study
5	LA1	America	United States, California, Los Angeles	**A1a1a1a1a**	MH587191	5	This study
6	LA2	America	United States, California, Los Angeles	**A1a1a1a1a**	MH587192	5	This study
7	LA3	America	United States, California, Los Angeles	**A1a1a1a1a**	MH587193	5	This study
8	LA8	America	United States, California, Los Angeles	**A1a1a1a1a**	MH587194	5	This study
9	LA11	America	United States, California, Los Angeles	**A1a1a1a1a**	MH587195	5	This study
10	LA4	America	United States, California, Los Angeles	**A1a1a1a1a**	MH587196	5	This study
11	VB3	America	United States, Florida, Vero Beach	**A1a1a1a1**	MH587197	7	This study
12	VB4	America	United States, Florida, Vero Beach	**A1a1a1a1**	MH587198	7	This study
13	Vir1	America	United States, Virginia	**A1a1a1a1b1**	KX383917	6	Battaglia et al. (2016)
14	Rc1	Europe	Italy, Reggio Calabria	**A1a1a1a1b1**	KX383918	6	Battaglia et al. (2016)
15	Vir2	America	United States, Virginia	**A1a1a1a1b1**	KX383919	6	Battaglia et al. (2016)
16	Ces1	Europe	Italy, Cesena	**A1a1a1a1b1**	KX383920	6	Battaglia et al. (2016)
17	CRM4^c^ ^,^ ^e^	Europe	Italy, Crema	**A1a1a1a1b1**	MH587217	6	This study
18	Cas1	Europe	Italy, Cassino	**A1a1a1a1b**	KX383921	6	Battaglia et al. (2016)
19	Pav3	Europe	Italy, Pavia	**A1a1a1a1b**	KX383922	6	Battaglia et al. (2016)
20	PoMo2600^d^	Europe	Portugal, Oporto	A1a1a1a1b	MN513353	N.D.	Zè-Zè et al. (2020)
21	PoMo2601^d^	Europe	Portugal, Oporto	A1a1a1a1b	MN513354	N.D.	Zè-Zè et al. (2020)
22	PoMo2604^d^	Europe	Portugal, Oporto	A1a1a1a1b	MN513356	N.D.	Zè-Zè et al. (2020)
23	PoMo2608^d^	Europe	Portugal, Oporto	A1a1a1a1b	MN513358	N.D.	Zè-Zè et al. (2020)
24	Mex1^e^	America	Mexico, Tapachula	**A1a1a1a1b**	MH587199	6	This study
25	J-Fu1^e^	Asia	Japan, Fukushima	**A1a1a1a1b**	MH587200	6	This study
26	J-Wa2	Asia	Japan, Wakayama	**A1a1a1a**	MH587201	5	This study
27	J-Wa3	Asia	Japan, Wakayama	**A1a1a1b**	MH587202	4	This study
28	J-Wa1	Asia	Japan, Wakayama	**A1a1a1b**	KX809765	4	Battaglia et al. (2016)
29	Cu1	Europe	France, Cuers, Var, PACA	**A1a1a1b**	MH587203	4	This study
30	PoMo2728^d^	Europe	Portugal, Algarve	A1a1a1b	MN513362	N.D.	Zè-Zè et al. (2020)
31	PoMoF636^d^	Europe	Portugal, Algarve	A1a1a1b	MN513368	N.D.	Zè-Zè et al. (2020)
32	Ces2	Europe	Italy, Cesena	**A1a1a**	KX383923	4	Battaglia et al. (2016)
33	CC1	Europe	Italy, Perugia	A1a1b	MH587204	5	This study
34	Cu4	Europe	France, Cuers, Var, PACA	A1a1b	MH587219	5	This study
35	PoMo2599^d^	Europe	Portugal, Algarve	A1a1b	MN513352	N.D.	Zè-Zè et al. (2020)
36	PoMo2711^d^	Europe	Portugal, Algarve	A1a1b	MN513361	N.D.	Zè-Zè et al. (2020)
37	PoMo2708^d^	Europe	Portugal, Algarve	A1a1b	MN513359	N.D.	Zè-Zè et al. (2020)
38	Cu2	Europe	France, Cuers, Var, PACA	A1a1b1a	MH587205	4	This study
39	Rim4^c^	Europe	Italy, Rimini	**A1a1b1a**	KX383929	4	Battaglia et al. (2016)
40	Tir1	Europe	Albania, Tirana	**A1a1b1a**	KX383930	4	Battaglia et al. (2016)
41	Tir2	Europe	Albania, Tirana	**A1a1b1a**	KX383931	4	Battaglia et al. (2016)
42	—	Asia	China, Jiangsu, Nanjing	**A1a1b1a**	KR068634	4	Zhang et al. (2015)
43	Ath2	Europe	Greece, Athens	A1a1b1	KX383932	4	Battaglia et al. (2016)
44	PoMo2607^d^	Europe	Portugal, Oporto	A1a1b1	MN513357	N.D.	Zè-Zè et al. (2020)
45	Pav4	Europe	Italy, Pavia	**A1a1b1**	KX383933	4	Battaglia et al. (2016)
46	Co1	Europe	Italy, Reggio Emilia	A1a1b1	MH587206	4	This study
47	Fo2^c^	Asia	China, Foshan	**A1a1b2a**	KX383934	4	Battaglia et al. (2016)
48	Fo4^c^	Asia	China, Foshan	**A1a1b2a**	MH587207	N.D.	This study
49	Fo1^c^	Asia	China, Foshan	**A1a1b2a**	MH587220	4	This study
50	Fo5^c^	Asia	China, Foshan	**A1a1b2a**	MH587221	4	This study
51	FPA1	Asia	China, Foshan	**A1a1b2a**	MH587222	4	This study
52	FPA2	Asia	China, Foshan	**A1a1b2a**	MH587223	4	This study
53	FPA3	Asia	China, Foshan	**A1a1b2a**	MH587224	4	This study
54	PoMo2602^d^	Europe	Portugal, Oporto	A1a1b2	MN513355	N.D.	Zè-Zè et al. (2020)
55	PoMoF505^d^	Europe	Portugal, Oporto	A1a1b2	MN513364	N.D.	Zè-Zè et al. (2020)
56	Cu5	Europe	France, Cuers, Var, PACA	**A1a**	MH587218	4	This study
57	PoMoF506^d^	Europe	Portugal, Algarve	A1a	MN513365	N.D.	Zè-Zè et al. (2020)
58	PoMoF607^d^	Europe	Portugal, Algarve	A1a	MN513366		Zè-Zè et al. (2020)
59	RdJ1	America	Brazil, Rio de Janeiro	**A1b1**	MH587208	4	This study
60	RdJ2	America	Brazil, Rio de Janeiro	**A1b1**	MH587209	4	This study
61	RdJ4	America	Brazil, Rio de Janeiro	**A1b1**	MH587211	4	This study
62	RdJ3	America	Brazil, Rio de Janeiro	**A1b1**	MH587210	4	This study
63	Lam2	Asia	Thailand, Lampang, Hang Chat	**A1b2a**	KX383925	3	Battaglia et al. (2016)
64	Ban7	Asia	Thailand, Uthai Thani, Ban Rai	**A1b2a**	KX383926	3	Battaglia et al. (2016)
65	Ath1	Europe	Greece, Athens	**A1b2a1**	KX383927	3	Battaglia et al. (2016)
66	Cam2^e^	Africa	Cameroon	**A1b2a1**	MH587212	3	This study
67	Cam3^e^	Africa	Cameroon	**A1b2a1**	MH587213	3	This study
68	VB1	America	United States, Florida, Vero Beach	**A1b2a1**	MH587214	4	This study
69	Cam1^e^	Africa	Cameroon	**A1b2b**	MH587215	5	This study
70	Cam4^e^	Africa	Cameroon	**A1b2b**	MH587216	5	This study
71	Los1	Asia	Philippines, Laguna, Los Baños	A2a	KX383935	3	Battaglia et al. (2016)
72	Los2	Asia	Philippines, Laguna, Los Baños	A2a	KX809761	3	Battaglia et al. (2016)
73	Los3	Asia	Philippines, Laguna, Los Baños	A2a	KX809762	3	Battaglia et al. (2016)
74	Los5	Asia	Philippines, Laguna, Los Baños	A2a	KX809764	3	Battaglia et al. (2016)
75	Los4	Asia	Philippines, Laguna, Los Baños	A2	KX809763	3	Battaglia et al. (2016)
76	—	Asia	Taiwan, Taipei	A3	NC006817	4	—

a ID numbers correspond to those in Figure 1.

b N.D., not determined.

c Laboratory-maintained strain.

d All sequences from Portugal (Zè-Zè et al., 2020) are from np 283 to np 14702.

e These samples were Sanger sequenced.

### DNA extraction, amplification and sequencing of *Ae. albopictus* mtDNAs

Genomic DNA from *Ae. albopictus* specimens was extracted from whole insect bodies, freshly collected or preserved in 80% ethanol at −20°C until extraction, with the Wizard^®^ Genomic DNA Purification Kit (Promega) following the manufacturer’s standard protocol. Each mitogenome was amplified in two partially overlapping long range PCR fragments, L1 and L2 (Battaglia et al., 2016), covering the coding region comprised between nps 274–14717 (NC006817), whereas the coding-region segments between nps 1–273 and nps 14718–14893 (NC006817) and the entire control (AT-rich) region (nps 14894–16665; NC006817) were amplified in two short PCR fragments, S1 and S2, using previously reported primer sets (Battaglia et al., 2016) and amplification protocols (Battaglia et al., 2016).

Most of the samples were sequenced using a Next Generation Sequencing (NGS) platform (MiSeq system, Illumina) with the MiSeq Reagent Nano Kit, v2 (300 cycles). The sequencing library was set-up using the Nextera XT DNA sample preparation kit (Illumina), following the manufacturer’s protocol, starting from the two PCR products L1 and L2 (∼0.75 ng of each PCR product), purified with Wizard^®^ SV Gel and PCR Clean-Up System (Promega) and quantified with a Quantus Fluorometer (Promega). On-board software created results in FASTQ format. Reads depth was generally >100X per each nucleotide position. The software Geneious v.8.1 (Kearse et al., 2012) was used to align FASTQ files to the tiger mosquito mitogenome #1 (KX383916, Figure 1) (Battaglia et al., 2016) obtained from the Italian Rimini strain (Bellini et al., 2007; Manni et al., 2015) in order to generate a single contig and a report of the sequence variants (nucleotide substitutions and indels). The threshold used for heteroplasmic positions was 20% and the average reads depth was ∼4,000X.

**FIGURE 1:**
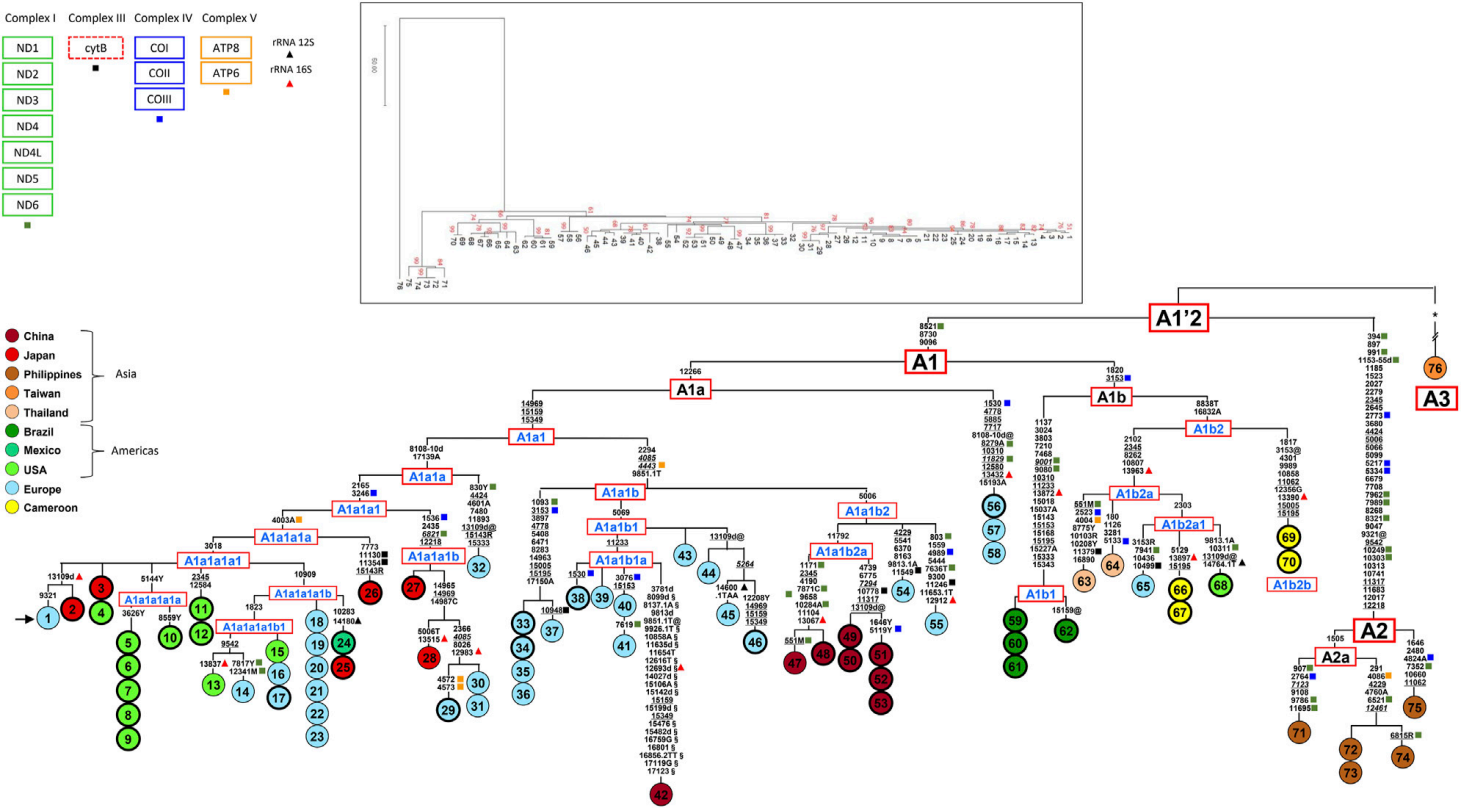
Phylogeny of *Ae. albopictus* mitogenomes. The tree encompasses 37 novel (circles marked by a thicker line) and 39 previously published sequences (24 from Battaglia et al., 2016, 1 from Zhang et al., 2015, 14 from Zé-Zé et al., 2020). The asterisk (*) refers to the 363 mutations (347 and 16 in the coding- and control-regions, respectively), listed in Supplementary Figure S1 (Battaglia et al., 2016), separating mitogenome #76 from the A1’2 node. For the phylogeny construction, the entire coding-region variation of all mitogenomes was included as well as some control-region mutations (from np 14897 to np 15350 and from np 16831 to np17150). The mitogenome from a mosquito of the Italian Rimini strain (#1, marked by the arrow) was employed to number the mutations shown on the branches. Mutations are transitions, unless a base is appended to indicate a transversion (to A, G, C, or T), or a suffix for indels (1, d). Heteroplasmic positions are indicated using the IUPAC nucleotide code. Recurrent mutations within the phylogeny are underlined (and in italics if present in mitogenome #76) and back mutations are marked with the suffix @. The numerous mutations shared only by the published mitogenomes #42 and #76 are marked with the suffix §. Sequences from Portugal (Zé-Zé et al., 2020) are from np 283 to np 14702; mutations out of this range were inferred according to their position in the tree. Colours illustrate geographic origins. Length variation (insertions/deletions) in polynucleotide stretches beginning at nps 3808, 8134, and 17140 were not considered. Haplogroups in blue are new relative to Battaglia et al., 2016. Non-synonymous mutations are indicated with squares, with colours matching the protein-coding gene complexes (top left in the figure); mutations in 12S and 16S rRNA genes are shown too, with blue and red diamonds. The MP tree is shown in the top inset; branch lengths are not proportional to molecular divergence.

The NGS method was used to sequence the two long range PCR fragments (L1 and L2), which covered almost the entire mitogenome coding-region. The missing coding-region segments (nps 1–273 and nps 14723–14896, KX383916) were Sanger sequenced, starting from the two short PCR fragments S1 and S2, and were assembled to the consensus sequence obtained in Geneious 8.1 with the software Sequencher 5.0 (Gene Codes Corporation), using mitogenome #1 as reference. A subset of the mitogenomes (i.e., #17, 24, 25, 66, 67, 69 and 70 in Table 1) were completely Sanger sequenced following a well-established protocol (Battaglia et al., 2016). Also in this case, they were aligned, assembled and compared to mitogenome #1, using Sequencher 5.0. The 37 *Ae. albopictus* mtDNA coding regions were submitted to GenBank (accession numbers MH587188–MH587224).

### Phylogeny construction and phylogeographic analyses

A Maximum Parsimony (MP) tree encompassing a total of 76 mitogenomes, including 37 novel sequences from this study, 37 previously published (Battaglia et al., 2016; Zé-Zé et al., 2020), the reference sequence from Taiwan (NC006817) and one sequence from the Jiangsu Province (China) (Zhang et al., 2015), was built using mitogenome #1 as reference for numbering nucleotide positions. Two of the previously published mitogenomes (#9 and #13; Battaglia et al., 2016) were not included because of gaps in their sequences.

The tree structure was also assessed using MEGA11 (Tamura et al., 2021) employing the Tree-Bisection-Regrafting (TBR) algorithm (Nei and Kumar, 2000) with default parameters for a MP reconstruction and a number of 1000 bootstrap replications (Figure 1). To conduct this analysis, we aligned the 76 coding-region sequences by performing a Multiple-Sequence Alignment with the Clustal algorithm (Chenna et al., 2003) implemented by Sequencher 5.0. Mitogenomes taken from Battaglia et al., 2016 are 23, two are previously published Taiwanese and Chinese sequences (NC006817 and KR068634) (Chen et al., 2015), 14 are from Zè-Zè et al., 2020 and 37 from this study (only one of the three FPA mitogenomes was considered).

A Bayesian tree was also inferred using BEAST1.10.4 (Drummond and Rambaut, 2007), under the HKY substitution model (gamma-distributed rates plus invariant sites) and running 50,000,000 iterations, with samples drawn every 10,000 Markov chain Monte Carlo (MCMC) steps. It was visualized using FigTree v.1.4.2. Phylogeny reconstruction was performed considering all the nucleotide substitutions (excluding indels and heteroplasmies) in the coding region (from np 1 to np 14896, relative to mitogenome #1) and the five informative control-region mutations 14969, 15159, 15349, 16832A, and 17139A.

To reconstruct the spatial dynamics of the tiger mosquito across the world, we performed a continuous phylogeographic analysis with BEAST 1.10.4, as described by (Dellicour et al., 2021). We used the same alignment of the phylogenetic reconstruction; sampling dates and coordinates were associated to each sample and Bayesian SkyGrid Coalescence model was set up under the HKY substitution model. We summarized the information within the sampled trees with TreeAnnotator, while the continuous phylogeographic analysis was visualized with SpreaD3 (Bielejec et al., 2016).

To evaluate population size trends through time, we obtained a Bayesian Skyline Plot (BSP) from BEAST 1.10.4 under the same model used for phylogeny reconstruction and considering a mutation rate calculated with Maximum Likelihood (ML) analysis. PamlX 1.3.1 (Yang 1997) was used to obtain ML estimates, assuming the HKY85 mutation model with gamma-distributed rates (approximated by a discrete distribution with 32 categories). Mutational distances were converted into years assuming an age of 32 years for haplogroup A1a1a1a1 (Bonizzoni et al., 2013) as a prior. This age was attributed taking into account that 1) this haplogroup most likely expanded first into North America and then into Europe; 2) *Ae. albopictus* was first detected in the United States (Port of Houston) in 1985; and iii) most of the samples were collected in 2017. We visualized the BSP obtained in plot with Tracer v1.5 and then converted it to an Excel graph by using a generation time that was twice (40 days), because of diapause in temperate regions, of that previously reported (20 days) (Nur Aida et al., 2008) for populations living in tropical areas.

## Results and discussion

To improve knowledge of *Ae. albopictus* mitogenome variation and acquire more information on the geographic sources of the recently established adventive populations, 37 novel mitogenomes were sequenced, many from areas not previously assessed (Battaglia et al., 2016; Zé-Zé et al., 2020). Table 1 reports the geographic origins of the 76 *Ae. albopictus* samples included in our study as well as their haplogroup/sub-haplogroup affiliations, which were re-defined, in some cases, on the basis of our more comprehensive phylogenetic survey.

### The updated mitogenome phylogeny of *Ae. albopictus*

The 37 novel mitogenomes were all from adventive *Ae. albopictus* populations, and all clustered into haplogroup A1. The lack of A2 or A3 mitogenomes confirms A1 as the haplogroup involved in the worldwide spread of the species. Their inclusion in the global *Ae. albopictus* mitogenome phylogeny allowed for the identification of a rather large number of previously unidentified sub-branches within A1, improving knowledge of their geographic distributions. As shown in MP tree of Figure 1 and the Bayesian tree of Supplementary Figure S1, haplogroup A1 comprises two major sister clades, A1a and A1b, which in turn are subdivided into numerous sub-branches. The overall tree structure is virtually identical with the two approaches, indicating high degree of internal consistency for all major branches.

Regarding A1a, the newly sequenced mitogenome #56 from Southern France bears the same haplotype as two Portuguese mitogenomes (#57–58) from Algarve (Figure 1). This shared haplotype redefines the distinguishing mutations of the node from which A1a departs, which is now solely defined by the transition at nucleotide position (np) 12266. Haplogroup A1a1 is further subdivided into A1a1a and A1a1b (formerly A1a1 and A1a2 in Battaglia et al., 2016). Within haplogroup A1a1a, two mitogenomes from Japan (#27–28) and three from Europe (#29–31) form a new well-defined clade, here named A1a1a1b, characterized by the mutational motif 1536–2435–6821–12218. The introduction of new samples from Japan and Mexico modified the former node A1a1a1 (Battaglia et al., 2016), and the haplogroup affiliation of the previously published samples descending from that node. The original (A1a1a1) node is now split into an ancestral node defined solely by the transversion T to A at np 4003, which includes the mitogenome from Wakayama #26 (Japan) as well as the rather common derived clade (A1a1a1a1) marked by the transition at np 3018. The latter includes the mitogenomes from Italy, Portugal and the United States previously classified as A1a1a1 (Battaglia et al., 2016; Zé-Zé et al., 2020) as well as 14 of the novel mitogenomes: three from different regions of Japan (#2, 3 and 25), five from California (#5–10), three from Florida (#4, 11 and 12), one from Mexico (#24) and the mitogenome from the Italian Crema strain (#17). The updated phylogenetic tree also permitted the identification of two major sub-haplogroups within A1a1a1a1, named A1a1a1a1a and A1a1a1a1b, the latter further split into two novel sub-branches.

The nomenclature of haplogroup A1a2 (Battaglia et al., 2016), now re-named as A1a1b, was also updated. It encompasses samples from Europe, mostly collected in Mediterranean countries (Albania, Greece, Italy, Portugal and Southern France) as well as from China. The Chinese samples form a sub-haplogroup (A1a1b2a) made up of four haplotypes, all found in the Foshan strain: the virtually identical mitogenomes #47 (Battaglia et al., 2016) and #48, which differ only by a segregating heteroplasmic variant, and mitogenomes #49–53, which form a sister branch with two haplotypes differing for two segregating heteroplasmic mutations. As expected, the three mitogenomes (#51–53) from the Foshan inbred strain, Pavia A, harbored the same haplotype. These findings indicate that at least two females, with distinct but related A1a1b2a mitogenomes, founded the Foshan strain.

Haplogroup A1b is split into A1b1 and A1b2. The first comprises only mitogenomes from Brazil, either identical (#59–61) or differing for a single reversion (#62), sharing a mutational motif of ten coding-region mutations, which include the transition at np 7210 in *ND5*, already identified as distinguishing marker for Brazilian *Ae. albopictus* (Birungi and Munstermann, 2002; Battaglia et al., 2016; Vega-Rùa et al., 2020). The composition of A1b2 is more geographically heterogeneous, since it encompasses the four samples (#66–67 and #69–70) from Africa (Cameroon), which are characterized by two divergent haplotypes within the branches A1b2a and A1b2b, both newly defined here.

### Tracing the recent spread of *Ae. albopictus* from its native range to new temperate and tropical areas

The 37 novel mitogenomes included in this study cluster into haplogroup A1, thus confirming this haplogroup as the only one involved in the worldwide spread of *Ae. albopictus*. It should be noted that A1 encompasses 70 of the 76 mitogenomes included in our phylogeny (Figure 1), but is made up by only 42 distinct haplotypes, with some differing from each other by only one or a few heteroplasmic variants, thus indicating the extremely recent occurrence of the distinguishing mutation(s).

The inclusion of the novel mitogenomes greatly improved the detail of the global mitogenome phylogeny, uncovering numerous previously unidentified branches and sub-branches, in particular within haplogroups A1a1a, A1a1b and A1b, thereby extending and improving previous results and conclusions.

It should be underlined that many of the tiger mosquitoes analyzed here (25 out of 37) were sampled in geographic areas that were not represented in previous studies, in particular California, Florida, Mexico, France, Cameroon, and different regions of Italy and Japan. This led to a better description of the geographic distributions of both the already identified haplogroups and the new ones.

### Temperate areas

**FIGURE 2:**
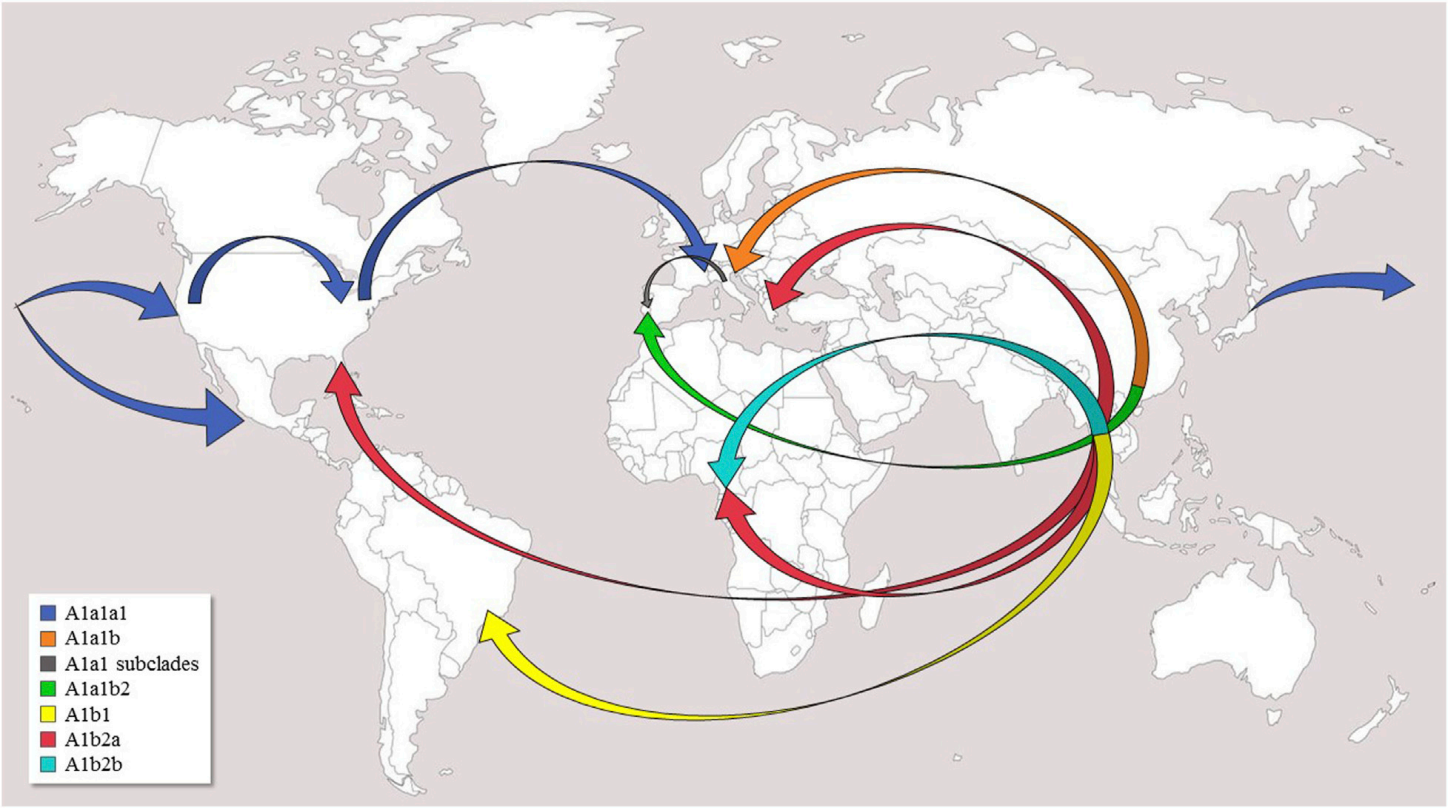
Possible worldwide diffusion routes of *Ae. albopictus*. Arrows indicate postulated diffusion routes from the native home-range (South-East Asia) and subsequent dispersals of major clades and subclades within haplogroup A1. The original world map is from the website (http://www.freeworldmaps.net).

Haplogroup A1a encompasses the majority of the mitogenomes (#1–55) in our phylogeny and is now present in the temperate areas of all continents (Figure 1). The identification of many previously unknown branches and sub-branches within haplogroup A1a allows new inferences to be made concerning the Asian sources of adventive populations and the migration routes followed by the mosquitoes that founded these recently established populations (Figure 2). A1a1a1 (mitogenomes #1–31), which has been detected in Europe, North America and Asia, is likely to have begun its spread from Japan. Indeed, the six Asian A1a1a1 haplotypes are all from Japan, and some are identical, or extremely closely related, to those found in Europe and North America. The spread of A1a1a1 involved at least four females, as attested by the four different subclades bearing Japanese mitogenomes, which might have originated from different regions of Japan. The out-of-Asia spread of these mitogenomes probably followed a Pacific route, initially reaching the coastal areas of California and western Mexico, then the eastern coast of the United States (Virginia and Florida, haplogroup A1a1a1a1), and finally Europe (Italy, France, Portugal).

Previous data had already identified genetic links between Japanese, North American and Italian populations, suggesting a Japanese source for the spread of *Ae. albopictus* to North America and later to Italy (Kambhampati et al., 1991; Urbanelli et al., 2000; Battaglia et al., 2016; Manni et al., 2017; Vega-Rùa et al., 2020), but our observations on the different subclades of A1a1a1 add further details to the overall picture. For instance, our Californian samples from Los Angeles all clustered into sub-haplogroup A1a1a1a1a, highlighting a single Japanese source. This result partially confirms previous data suggesting diversified Asian sources (Japan, South China, Singapore) for Californian tiger mosquito populations (Zhong et al., 2013).

A new important piece of information is provided by the single Mexican mitogenome (#24), which belongs to A1a1a1a1b. It harbors a sequence identical to the Japanese mitogenome #25. When considering that the Mexican mosquito was collected in Tapachula, a major trading site in the far south of the Mexican Pacific coast, this finding suggests that *Ae. albopictus* was introduced into Mexico either by land, from the United States (Pech-May et al., 2016; Vega-Rùa et al., 2020), or by sea directly from a Japanese source.

Another route of spread is possibly marked by the distribution of A1a1b, the sister branch of A1a1a (Figure 2). Taking into account that the A1a1b mitogenomes found in Europe cluster into at least four rather divergent subsets (Figure 1), and that so far all Asian members of A1a1b are from China, the most parsimonious scenario, at least with currently available data, is that A1a1b arose in China, possibly from multiple geographic sources, and from there it directly reached Mediterranean Europe (Albania, Greece, Italy, Southern France, and Portugal). However, an additional intermediate step through La Rèunion (as often suggested, Vega-Rùa et al., 2021) cannot be ruled out based on our phylogeny, since it lacks mitogenomes from this geographic area, which played a pivotal role in the demographic history of *Ae. albopictus*. Our data depict a scenario in which the Mediterranean basin was affected by multiple invasions of the tiger mosquito from different geographic areas and at different times (Vega-Rùa et al., 2021). In particular, our findings are in agreement with the scenario that Italian tiger mosquitoes have at least a dual origin: from North America, as discussed above for A1a1a, and from an eastern Asian source, although indirectly, i.e., reaching Italy possibly through other Mediterranean sources such as Albania and/or Greece, both being among the most important sea-trading partners with China (Enserink, 2008; Battaglia et al., 2016).

Also, the inclusion of four specimens from southeastern France further details the worldwide spread of *Ae. albopictus*. They were all collected in Cuers, close to the beaches of Hyère, in the Provence-Alpes-Côte d’Azur, between Toulon (a cargo port and French naval base) and Marseille, the second busiest port of the Mediterranean Sea, and they harbored four distinct haplotypes, falling into haplogroups A1a1a1b (#29), A1a1b (#34), A1a1b1a (#38), and A1a, which directly descends from the root (#56). Our phylogeny takes the Portuguese samples from Zé-Zè et al., 2020 into consideration as well. The first detection of tiger mosquitoes in Portugal dates back to no earlier than 2017, when most of the other Mediterranean countries had already been invaded. The Portuguese haplotypes fall within haplogroup A1a1 variability, belonging to at least seven different subclades. The overall phylogeography of haplogroup A1a1 points to multiple independent introduction events in both France and Portugal, from different geographic regions, via human-mediated transports. Some events were promoted by land transportations, mainly from Italy (as already suggested by Zé-Zé et al., 2020), probably passing through Southern France, towards both the Algarve and Oporto regions, as marked by the A1a1a1a1b (#20–23), A1a1a1b (#30–31), A1a1b (#35–37) and A1a1b1 (#44) haplotypes, and by the two mitogenomes from Algarve descending from the A1a root (#57–58) bearing the same haplotype of the previously mentioned French mitogenome #56. Other introduction events appear instead to have involved international connections through seaports. This seems particularly true for the Oporto region, where a direct link with China is testified by haplogroup A1a1b2, which for the moment includes only Chinese and Oporto mitogenomes (#47–55). However, our phylogeny lacks mitogenomes from Spain, a key geographic region whose inclusion could be vital for better depicting the geographic spread of this invasive species in Iberia. Overall, this pattern strongly hints to the role of both Mediterranean ports and land trade routes in the mosquito’s spread within the context of worldwide trade.

In brief, it appears that human-mediated trade activities triggered the diffusion of different A1a subclades in worldwide temperate regions. Thus, the model of this invasive species’ spread seems now consolidated, based on long inter-continental distances covered by sea-trade, followed by localized spread by land around cities where goods arriving from Asia are sorted and redirected for further distribution.

### Tropical and sub-tropical areas

With the exception of one mitogenome (#65) from Athens (Greece), the two A1b sub-branches (A1b1 and A1b2) exclusively encompass samples from either tropical (Brazil, Thailand, Cameroon) or sub-tropical (Florida) areas (Figure 1).

A1b1 is made up exclusively of the four Brazilian mitogenomes from Rio de Janeiro, an urban context with a tropical climate. In the refined phylogeny they cluster tightly, virtually as a single haplotype. The mutational motif of this branch includes the transition at np 7210 in the *ND5* gene, which was often surveyed in early studies. This mutation was previously reported as a marker of Brazilian *Ae. albopictus* (Birungi and Munstermann, 2002) and identified also in two other *ND5* published haplotypes: one from Phuntsholing in Southern Bhutan (JQ436953) and one from Chiang Mai in Thailand (JQ436956) (Porretta et al., 2012; Battaglia et al., 2016). These findings suggest that Indochina might be the ancestral source of the A1b1 mitogenomes found in Brazil, pointing to a possible direct connection between the two geographic areas (Battaglia et al., 2016; Goubert et al., 2016). This observation is in contrast with the very first allozyme results (Kambhampati et al., 1991) claiming that both Brazilian and United States tiger mosquitoes had originated in Japan, suggesting, instead, separate introductions in Brazil and United States, from different sources (Birungi and Munstermann, 2002; Vega-Rùa et al., 2020). Such a scenario of distinct introduction events is supported also by the ability to induce diapause, absent in tropical areas (such as Indochina and Brazil) and present in temperate areas (such as Japan and United States) (Batz et al., 2020).

In contrast, A1b2 is extremely well-differentiated in terms of both haplotype variation and geographical distribution (Figure 1). Indeed, its sub-clades A1b2a and A1b2b include samples from geographically distant tropical countries (Cameroon and Thailand), the mitogenome (#65) from Athens and one (#68) from Florida, in temperate and sub-tropical contexts, respectively. The phylogenetic links between these mitogenomes suggest Thailand as the Asian ancestral source, or at least an intermediate one, for the now worldwide-diffused A1b2a branch. However, neighboring countries in Indochina are also strong candidates. The sub-branch A1b2a1 links closely the Greek and Florida samples, as well as two from Cameroon (#66 and #67, with the same haplotype). It should be underlined that Port Piraeus in Athens is a hub for shipping from Asia, including Thailand, to the Mediterranean region (Manni et al., 2017; Vega-Rùa et al., 2020).

An additional recent introduction in Cameroon can be hypothesized for mitogenomes #69 and #70, again with the same haplotype, which form A1b2b, whose very distinctive mutational motif has not been found elsewhere. However, a previous survey of the *COI* variation in 259 *Ae. albopictus* from Australasia, South-East Asia, islands of the Indian and Pacific Oceans as well as the United States (Maynard et al., 2007) revealed ten samples harboring the transitions at nps 1820 and 1817, located at the A1b and A1b2b nodes, respectively (Figure 1). These two transitions are part of the mutational motif shared by the Cameroonian mitogenomes #69 and #70. Interestingly, eight of the ten *COI* sequences harboring both mutations are from Malaysia (haplotype accession numbers KY907368-69 from Kuala Lumpur; KY907370-71 from Ipoh) and two from Singapore (haplotype accession number KY907372), thus strongly favoring the hypothesis that the Malaysian Peninsula was the second source of Cameroon tiger mosquitoes.

The overall scenario marked by the A1b sub-branches suggests that (a) haplogroup A1b2 derives from an Asian tropical area that definitely includes Thailand and Malaysia, but other as-yet unsurveyed geographical sources in Indochina are also possible; (b) the current (and limited) distribution of A1b2 in adventive populations might be restrained by climate conditions. Indeed, outside tropical areas, we have found A1b2 mitogenomes only in Athens (Greece) and Florida, both with climate conditions that are indeed quite close to those of sub-tropical areas.

### Reconstruction of the dispersal history through a continuous phylogeographic analysis

To further assess the trajectory of tiger mosquito across its current distribution range, we also performed a continuous phylogeographic analysis (Dellicour et al., 2021), resulting from Bayesian molecular clock inference of sequence and trait evolutionary processes, by considering both sampling dates and coordinates as traits. The resulting dispersal map is depicted in Supplementary Figure S2. It confirms that 1) South East Asia is the native homeland of the tiger mosquito, as revealed by darker red shades indicating an older and prolonged presence in those geographic areas, and 2) supports the trajectory routes of Figure 2, which were obtained by taking into consideration historical data. However, it is worth mentioning that our samples were all collected in the last twelve years, despite tiger mosquitoes colonized the sampled regions earlier, in some cases several decades prior to twelve years ago, and at different times.

### Population size trend

To investigate *Ae. albopictus* population size trends, a Bayesian Skyline Plot (BSP) was also obtained (Supplementary Figure S3), assuming that haplogroup A1a1a1a1 expanded in North America and taking into account that *Ae. albopictus* was first recorded in continental United States in 1985. An initial episode of population expansion started at ∼120 years ago in agreement with available information concerning the first diffusion of this species outside its ancestral East Asian homeland, which is dated back at the beginning of the last century. However, it should be underlined that the poor representation of Asian samples in our data set does not allow to identify how many Asian founder haplotypes gave rise to the A1a1a1a1 branches detected in Europe and North America. Thus, the assumption of an age of 32 years for the expansion of A1a1a1a1 needs to be reassessed in the future when additional Asian mitogenomes will become available.

## Conclusion

The analysis of 37 novel mitogenomes of *Ae. albopictus* from numerous wild adventive populations and some laboratory strains, in the context of previously available data, allowed an in-depth refinement of the mitogenome phylogeny of *Ae. albopictus* to be extensively refined. This species is characterized by three major divergent haplogroups (A1, A2 and A3), but only haplogroup A1 with its numerous clades and sub-clades, largely re-defined in this study, is actively involved in the recent worldwide spread.

As expected in populations founded by a few females from the same geographic source, thus strongly affected by founder events and genetic drift, shared haplotypes characterize most if not all the mitogenomes deriving from the same lab strain, for instance mitogenomes #49–50 from the Chinese Foshan strain and mitogenomes #51–53 from the Foshan inbred strain Pavia A (FPA). However, the major role played by founder events in the current distribution of the tiger mosquito mitogenome variation is also clearly visible in many of the wild adventive populations. For instance, five of the six mosquitoes (#5–10) from Los Angeles (California) share the same haplotype, and the sixth (#10) harbors a closely related haplotype within the same local sub-haplogroup (A1a1a1a1a). A similar scenario, three identical mitogenomes and one differing by a single reversion, is highlighted by the four mitogenomes (#59–62) from Rio de Janeiro (Brazil), which form the highly divergent sub-haplogroup A1b1. However, in other cases the founder event is not restricted to a single country, but shared by multiple countries or even by distant continents, highlighting a series of clonal and sub-clonal founding events, which occurred sequentially and involved the same founder mitogenome or one of its immediate, often still heteroplasmic, derivatives. This observation also implies that the fast and extremely invasive spread of tiger mosquitoes often started and/or involved few individuals, at least from a female perspective, thus pointing to the crucial role of control strategies.

The connections between the haplotypes/branches detected in the different areas confirm the previously proposed scenario and provides fresh clues to the Asian sources of European, African and American adventive populations.

Haplogroup A1a harbors two main starting points, Japan (A1a1a) and China (A1a1b), and spread mainly along the temperate belt. From Japan, A1a1a most likely moved first to North America and from there to Europe, while A1a1b arrived directly in Europe from China. In contrast, A1b arose in the tropical areas of South East Asia, most likely Thailand and/or Malaysia. From there it expanded to the tropical areas of Africa (Cameroon), possibly with two separate invasion events, but also reached Greece and Florida, which are not in tropical areas, but are characterized by climatic conditions not too dissimilar to those of sub-tropical regions.

Our new insights into *Ae. albopictus* mitogenome variation could provide important information to control the current spread of this invasive species and limit its detrimental social, medical and economic effects.

Future studies should perform an assessment of the *Ae. albopictus* mitogenome sequence variation worldwide, also in comparison with both closely related and distant species, in order to identify candidate mtDNA mutations that might play a functional role and facilitate the spread of specific haplogroups into temperate regions. Moreover, functional evaluations/analyses involving living mosquitoes (from lab strains and possibly wild populations), cells or cellular extracts and larvae should be considered to confirm/dismiss the potential adaptive role of candidate mtDNA mutations.

Finally, further analyses are needed to clarify whether the extreme divergence of the single A3 mitogenome from all other mitogenomes is simply due to different arrival sources, or to local population divergences (Ruiling et al., 2018b) or to the presence in Taiwan of cryptic species, as already shown in some regions of China (Guo et al., 2018).

## Data Availability

The datasets presented in this study can be found in online repositories. The names of the repository/repositories and accession number(s) can be found in the article/Supplementary Material.

## References

[B1] Adhami J. Murati N. (1987). Presence du moustique *Aedes albopictus* en Albanie. Rev. Mjekesore 1, 13–16.

[B2] Battaglia V. Gabrieli P. Brandini S. Capodiferro M. R. Javier P. A. Chen X.-G. (2016). The worldwide spread of the tiger mosquito as revealed by mitogenome haplogroup diversity. Front. Genet. 7, 208. 10.3389/fgene.2016.00208 27933090 PMC5120106

[B3] Batz Z. A. Clemento A. J. Fritzenwanker J. Ring T. J. Garza J. C. Armbruster P. A. (2020). Rapid adaptive evolution of the diapause program during range expansion of an invasive mosquito. Evolution 74, 1451–1465. 10.1111/evo.14029 32490563 PMC8023039

[B4] Beebe N. W. Ambrose L. Hill L. A. Davis J. B. Hapgood G. Cooper R. D. (2013). Tracing the tiger: Population genetics provides valuable insights into the *Aedes* (stegomyia) *albopictus* invasion of the australasian region. PLoS Negl. Trop. Dis. 7, e2361. 10.1371/journal.pntd.0002361 23951380 PMC3738475

[B5] Bellini R. Calvitti M. Medici A. Carrieri M. Celli G. Maini S. (2007). “Use of the sterile insect technique against *Aedes albopictus* in Italy: First results of a pilot trial,” in Use of the sterile insect technique against *Aedes albopictus* in Italy: First results of a pilot trial” in area-wide control of insect pests. Editors Vreysen M. J. B. Robinson A. S. Hendrichs J. (Dordrecht, Netherlands: Springer), 505–515. 10.1007/978-1-4020-6059-5_47

[B6] Benedict M. Q. Levine R. S. Hawley W. A. Lounibos L. P. (2007). Spread of the tiger: Global risk of invasion by the MosquitoAedes albopictus. Vector-Borne Zoonotic Dis. 7, 76–85. 10.1089/vbz.2006.0562 17417960 PMC2212601

[B7] Benelli G. Wilke A. B. B. Beier J. C. (2020). *Aedes albopictus* (Asian tiger mosquito). Trends Parasitol. 36, 942–943. 10.1016/j.pt.2020.01.001 32037135

[B8] Bielejec F. Baele G. Vrancken B. Suchard M. A. Rambaut A. Lemey P. (2016). SpreaD3: Interactive visualization of spatiotemporal history and trait evolutionary processes. Mol. Biol. Evol. 33, 2167–2169. 10.1093/molbev/msw082 27189542 PMC6398721

[B9] Birungi J. Munstermann L. E. (2002). Genetic structure of *Aedes albopictus* (Diptera: Culicidae) populations based on mitochondrial ND5 sequences: Evidence for an independent invasion into Brazil and United States. an 95, 125–132. 10.1603/0013-8746(2002)095[0125:gsoaad]2.0.co;2

[B10] Bonizzoni M. Gasperi G. Chen X. James A. A. (2013). The invasive mosquito species *Aedes albopictus*: Current knowledge and future perspectives. Trends Parasitol. 29, 460–468. 10.1016/j.pt.2013.07.003 23916878 PMC3777778

[B11] Chen X.-G. Jiang X. Gu J. Xu M. Wu Y. Deng Y. (2015). Genome sequence of the Asian Tiger mosquito, *Aedes albopictus* , reveals insights into its biology, genetics, and evolution. Proc. Natl. Acad. Sci. U.S.A. 112, E5907–E5915. 10.1073/pnas.1516410112 26483478 PMC4640774

[B12] Chenna R. Sugawara H. Koike T. Lopez R. Gibson T. J. Higgins D. G. (2003). Multiple sequence alignment with the clustal series of programs. Nucleic Acids Res. 31, 3497–3500. 10.1093/nar/gkg500 12824352 PMC168907

[B13] da Silva A. F. Machado L. C. de Paula M. B. da Silva Pessoa Vieira C. J. de Morais Bronzoni R. V. de Melo Santos M. A. V. (2020). Culicidae evolutionary history focusing on the Culicinae subfamily based on mitochondrial phylogenomics. Sci. Rep. 10, 18823. 10.1038/s41598-020-74883-3 33139764 PMC7606482

[B14] Dellicour S. Gill M. S. Faria N. R. Rambaut A. Pybus O. G. Suchard M. A. (2021). Relax, keep walking - a practical guide to continuous phylogeographic inference with BEAST. Mol. Biol. Evol. 38, 3486–3493. 10.1093/molbev/msab031 33528560 PMC8321535

[B15] Dritsou V. Topalis P. Windbichler N. Simoni A. Hall A. Lawson D. (2015). A draft genome sequence of an invasive mosquito: An ItalianAedes albopictus. Pathogens Glob. Health 109, 207–220. 10.1179/2047773215Y.0000000031 PMC4727573 26369436

[B16] Drummond A. J. Rambaut A. (2007). Beast: Bayesian evolutionary analysis by sampling trees. BMC Evol. Biol. 7, 214. 10.1186/1471-2148-7-214 17996036 PMC2247476

[B17] Enserink M. (2008). A mosquito goes global. Science 320, 864–866. 10.1126/science.320.5878.864 18487167

[B19] European Centre For Disease Prevention and Control. (2022). Ecdc. Available online: http://Ecdc.Europa.Eu .

[B20] Fang Y. Zhang J. Wu R. Xue B. Qian Q. Gao B. (2018). Genetic polymorphism study on *Aedes albopictus* of different geographical regions based on DNA barcoding. BioMed Res. Int. 2018, 1–10. 10.1155/2018/1501430 PMC5996416 30003088

[B21] Gao J. Zhang H. D. Guo X. X. Xing D. Dong Y.-D. Lan C.-J. (2021). Dispersal patterns and population genetic structure of *Aedes albopictus* (Diptera: Culicidae) in three different climatic regions of China. Parasites Vectors 14 (1), 12. 10.1186/s13071-020-04521-4 33407824 PMC7789686

[B22] Global Invasive Species Database. (2010). Iucngisd. AvailableAt: http://Www.Iucngisd.Org/Gisd/100_worst.Php .

[B23] Goubert C. Minard G. Vieira C. Boulesteix M. (2016). Population genetics of the Asian tiger mosquito *Aedes albopictus*, an invasive vector of human diseases. Heredity 117, 125–134. 10.1038/hdy.2016.35 27273325 PMC4981682

[B24] Gratz N. G. (2004). Critical review of the vector status of *Aedes albopictus* . Med. Vet. Entomol. 18, 215–227. 10.1111/j.0269-283X.2004.00513.x 15347388

[B25] Guo Y. Song Z. Luo L. Wang Q. Zhou G. Yang D. (2018). Molecular evidence for new sympatric cryptic species of *Aedes albopictus* (Diptera: Culicidae) in China: A new threat from *Aedes albopictus* subgroup? Parasites Vectors 11, 228. 10.1186/s13071-018-2814-8 29618379 PMC5885320

[B26] Ibáñez-Justicia A. Smitz N. den Hartog W. van de Vossenberg B. De Wolf K. Deblauwe I. (2020). Detection of exotic mosquito species (Diptera: Culicidae) at international airports in Europe. Ijerph 17, 3450. 10.3390/ijerph17103450 PMC7277938 32429218

[B27] Ismail N.-A. Adilah-Amrannudin N. Hamsidi M. Ismail R. Dom N. C. Ahmad A. H. (2017). The genetic diversity, haplotype analysis, and phylogenetic relationship of *Aedes albopictus* (Diptera: Culicidae) based on the cytochrome oxidase 1 marker: A Malaysian scenario. J. Med. Entomol. 54, 1573–1581. 10.1093/jme/tjx126 28981849

[B28] Kambhampati S. Black W. C. Rai K. S. (1991). Geographic origin of the US and Brazilian *Aedes albopictus* inferred from allozyme analysis. Heredity 67, 85–94. 10.1038/hdy.1991.67 1917554

[B29] Kearse M. Moir R. Wilson A. Stones-Havas S. Cheung M. Sturrock S. (2012). Geneious basic: An integrated and extendable desktop software platform for the organization and analysis of sequence data. Bioinformatics 28, 1647–1649. 10.1093/bioinformatics/bts199 22543367 PMC3371832

[B30] Knudsen A. B. (1995). Global distribution and continuing spread of *Aedes albopictus* . Parassitologia 37, 91–97. 8778670

[B31] Kraemer M. U. G. Reiner R. C. Brady O. J. Messina J. P. Gilbert M. Pigott D. M. (2019). Past and future spread of the arbovirus vectors *Aedes aegypti* and *Aedes albopictus* . Nat. Microbiol. 4, 854–863. 10.1038/s41564-019-0376-y 30833735 PMC6522366

[B32] Kraemer M. U. G. Sinka M. E. Duda K. A. Mylne A. Shearer F. M. Brady O. J. (2015). The global compendium of *Aedes aegypti* and *Ae. albopictus* occurrence. Sci. Data 2, 150035. 10.1038/sdata.2015.35 26175912 PMC4493829

[B33] Lee E. Yang S.-C. Kim T.-K. Noh B.-E. Lee H. S. Kim H. (2020). Geographical genetic variation and sources of Korean *Aedes albopictus* (Diptera: Culicidae) populations. J. Med. Entomol. 57, 1057–1068. 10.1093/jme/tjz254 31972007

[B34] Manni M. Gomulski L. M. Aketarawong N. Tait G. Scolari F. Somboon P. (2015). Molecular markers for analyses of intraspecific genetic diversity in the Asian Tiger mosquito, *Aedes albopictus* . Parasites Vectors 8, 188. 10.1186/s13071-015-0794-5 25890257 PMC4404008

[B35] Manni M. Guglielmino C. R. Scolari F. Vega-Rúa A. Failloux A.-B. Somboon P. (2017). Genetic evidence for a worldwide chaotic dispersion pattern of the arbovirus vector, *Aedes albopictus* . PLoS Negl. Trop. Dis. 11, e0005332. 10.1371/journal.pntd.0005332 28135274 PMC5300280

[B36] Marconcini M. Pischedda E. Houé V. Palatini U. Lozada-Chávez N. Sogliani D. (2021). Profile of small RNAs, vDNA forms and viral integrations in late chikungunya virus infection of *Aedes albopictus* mosquitoes. Viruses 13, 553. 10.3390/v13040553 33806250 PMC8066115

[B37] Mariconti M. Obadia T. Mousson L. Malacrida A. Gasperi G. Failloux A.-B. (2019). Estimating the risk of arbovirus transmission in Southern Europe using vector competence data. Sci. Rep. 9, 17852. 10.1038/s41598-019-54395-5 31780744 PMC6882796

[B38] Maynard A. J. Ambrose L. Cooper R. D. Chow W. K. Davis J. B. Muzari M. O. (2007). Tiger on the prowl: Invasion history and spatio-temporal genetic structure of the asian tiger mosquito *Aedes albopictus* (skuse 1894) in the indo-pacific. PLoS Negl. Trop. Dis. 11, e0005546. 10.1371/journal.pntd.0005546 PMC5406021 28410388

[B39] Medlock J. M. Hansford K. M. Versteirt V. Cull B. Kampen H. Fontenille D. (2015). An entomological review of invasive mosquitoes in Europe. Bull. Entomol. Res. 105, 637–663. 10.1017/S0007485315000103 25804287

[B40] Nei M. Kumar S. (2000). Molecular evolution and phylogenetics. New York: Oxford University Press.

[B41] Neteler M. Roiz D. Rocchini D. Castellani C. Rizzoli A. (2011). Terra and aqua satellites track tiger mosquito invasion: Modelling the potential distribution of *Aedes albopictus* in North-eastern Italy. Int. J. Health Geogr. 10, 49. 10.1186/1476-072X-10-49 21812983 PMC3170180

[B42] Nur Aida H. Abu Hassan A. Nurita A. T. Che Salmah M. R. Norasmah B. (2008). Population analysis of *Aedes albopictus* (Skuse) (Diptera:Culicidae) under uncontrolled laboratory conditions. Trop. Biomed. 25, 117–125. 18948882

[B43] Palatini U. Masri R. A. Cosme L. V. Koren S. Thibaud-Nissen F. Biedler J. K. (2020). Improved reference genome of the arboviral vector *Aedes albopictus* . Genome Biol. 21, 215. 10.1186/s13059-020-02141-w 32847630 PMC7448346

[B64] Pasquali S. Mariani L. Calvitti M. Moretti R. Ponti L. Chiari M. (2020). Development and calibration of a model for the potential establishment and impact of Aedes albopictus in Europe. Acta Trop., 105228. 10.1016/j.actatropica.2019.105228 31678121

[B44] Pech-May A. Moo-Llanes D. A. Puerto-Avila M. B. Casas M. Danis-Lozano R. Ponce G. (2016). Population genetics and ecological niche of invasive *Aedes albopictus* in Mexico. Acta Trop. 157, 30–41. 10.1016/j.actatropica.2016.01.021 26814619

[B45] Porretta D. Mastrantonio V. Bellini R. Somboon P. Urbanelli S. (2012). Glacial history of a modern invader: Phylogeography and species distribution modelling of the asian tiger mosquito *Aedes albopictus* . PLoS ONE 7, e44515. 10.1371/journal.pone.0044515 22970238 PMC3435282

[B46] Roiz D. Neteler M. Castellani C. Arnoldi D. Rizzoli A. (2011). Climatic factors driving invasion of the tiger mosquito (*Aedes albopictus*) into new areas of Trentino, Northern Italy. PLoS ONE 6, e14800. 10.1371/journal.pone.0014800 21525991 PMC3078124

[B47] Roiz D. Ruiz S. Soriguer R. Figuerola J. (2014). Climatic effects on mosquito abundance in Mediterranean wetlands. Parasites Vectors 7, 333. 10.1186/1756-3305-7-333 25030527 PMC4223583

[B48] Ruiling Z. Peien L. Xuejun W. Zhong Z. (2018a). Molecular analysis and genetic diversity of *Aedes albopictus* (Diptera, Culicidae) from China. Mitochondrial DNA Part A 29, 594–599. 10.1080/24701394.2017.1325481 28502235

[B49] Ruiling Z. Tongkai L. Dezhen M. Zhong Z. (2018b). Genetic characters of the globally spread tiger mosquito,*Aedes albopictus*(Diptera, Culicidae): Implications from mitochondrial gene COI. J. Vector Ecol. 43, 89–97. 10.1111/jvec.12287 29757513

[B50] Sabatini A. Raineri V. Trovato G. Coluzzi M. (1990). *Aedes albopictus* in Italy and possible diffusion of the species into the Mediterranean area. Parassitologia 32, 301–304. 2132441

[B51] Schoener E. Zittra C. Weiss S. Walder G. Barogh B. S. Weiler S. (2019). Monitoring of alien mosquitoes of the genus Aedes (Diptera: Culicidae) in Austria. Parasitol. Res. 118, 1633–1638. 10.1007/s00436-019-06287-w 30877440 PMC6478629

[B52] Scholte E. J. Schaffner F. (2007). “Waiting for the tiger: Establishment and spread of the *Aedes albopictus* mosquito in Europe,” in Emerging pests and vector-borne diseases in Europe. Editors Takken W. Knols B. G. J. (The Netherlands: Wageningen Academic Publishers).

[B65] Skuse F. A. (1894). The banded mosquito of Bengal. Indian Museum Notes 3, 20. 10.5281/zenodo.163480

[B53] Șuleșco T. Bușmachiu G. Lange U. Schmidt-Chanasit J. Lühken R. (2021). The first record of the invasive mosquito species *Aedes albopictus* in Chişinӑu, Republic of Moldova, 2020. Parasites Vectors 14, 565. 10.1186/s13071-021-05060-2 PMC8565072 34732241

[B54] Tamura K. Stecher G. Kumar S. (2021). MEGA11: Molecular evolutionary genetics analysis version 11. Mol. Biol. Evol. 38, 3022–3027. 10.1093/molbev/msab120 33892491 PMC8233496

[B55] Tedjou A. N. Kamgang B. Yougang A. P. Njiokou F. Wondji C. S. (2019). Update on the geographical distribution and prevalence of *Aedes aegypti* and *Aedes albopictus* (Diptera: Culicidae), two major arbovirus vectors in Cameroon. PLoS Negl. Trop. Dis. 13, e0007137. 10.1371/journal.pntd.0007137 30883552 PMC6438584

[B56] Torroni A. Achilli A. Macaulay V. Richards M. Bandelt H. (2006). Harvesting the fruit of the human mtDNA tree. Trends Genet. 22, 339–345. 10.1016/j.tig.2006.04.001 16678300

[B57] Urbanelli S. Bellini R. Carrieri M. Sallicandro P. Celli G. (2000). Population structure of *Aedes albopictus* (skuse): The mosquito which is colonizing mediterranean countries. Heredity 84 (3), 331–337. 10.1046/j.1365-2540.2000.00676.x 10762403

[B58] Vega-Rúa A. Marconcini M. Madec Y. Manni M. Carraretto D. Gomulski L. M. (2020). Vector competence of *Aedes albopictus* populations for chikungunya virus is shaped by their demographic history. Commun. Biol. 3, 326. 10.1038/s42003-020-1046-6 32581265 PMC7314749

[B59] Yang Z. (1997). Paml: A program package for phylogenetic analysis by maximum likelihood. Bioinformatics 13, 555–556. 10.1093/bioinformatics/13.5.555 9367129

[B60] Zé-Zé L. Borges V. Osório H. C. Machado J. Gomes J. P. Alves M. J. (2020). Mitogenome diversity of *Aedes* (stegomyia) *albopictus*: Detection of multiple introduction events in Portugal. PLoS Negl. Trop. Dis. 14, e0008657. 10.1371/journal.pntd.0008657 32997656 PMC7549828

[B61] Zhang H. Xing D. Wang G. Li C. Zhao T. (2015). Sequencing and analysis of the complete mitochondrial genome of *Aedes albopictus* (Diptera: Culicidae) in China. Mitochondrial DNA Part A 27, 2787–2788. 10.3109/19401736.2015.1053067 26114325

[B62] Zhong D. Lo E. Hu R. Metzger M. E. Cummings R. Bonizzoni M. (2013). Genetic analysis of invasive *Aedes albopictus* populations in Los Angeles County, California and its potential public health impact. PLoS ONE 8, e68586. 10.1371/journal.pone.0068586 23861921 PMC3702605

[B63] Žitko T. Kovačić A. Desdevises Y. Puizina J. (2011). Genetic variation in east-adriatic populations of the asian tiger mosquito, *Aedes albopictus* (Diptera: Culicidae), inferred from NADH5 and COI sequence variability. Eur. J. Entomol. 108, 501–508. 10.14411/eje.2011.065

