# Supplementary material for: The worldwide spread of Aedes albopictus: New insights from mitogenomes
Source: Front Genet. 2022 Aug 26;13:931163. doi: 10.3389/fgene.2022.931163 (PMC9459080; doi:10.3389/fgene.2022.931163)
Supplement: Supplementary file 1 [file DataSheet1.DOCX]

**Supplementary Material**

**The worldwide spread of *Aedes albopictus*: new insights from mitogenomes**

Vincenza Battaglia^1†^, Vincenzo Agostini^1†^, Elisabetta Moroni^1^, Giulia Colombo^1^, Gianluca Lombardo^1^, Nicola Rambaldi Migliore^1^, Paolo Gabrieli^1,2^, Maria Garofalo^3^, Stella Gagliardi^3^, Ludvik M. Gomulski^1^, Luca Ferretti^1^, Ornella Semino^1^, Anna R. Malacrida^1^, Giuliano Gasperi^1^, Alessandro Achilli^1^, Antonio Torroni^1^, Anna Olivieri^1*^

^1^Dipartimento di Biologia e Biotecnologie "L. Spallanzani", Università di Pavia, Pavia, Italy.

^2^ Department of Biosciences and Pediatric Clinical Research Center "Romeo ed Enrica Invernizzi", University of Milan, Milan, Italy.

^3^ Molecular Biology and Transcriptomic Unit, IRCCS Mondino foundation, Pavia, 27100, Italy.

^†^These authors contributed equally to this work and share first authorship.

*Corresponding author: anna.olivieri@unipv.it


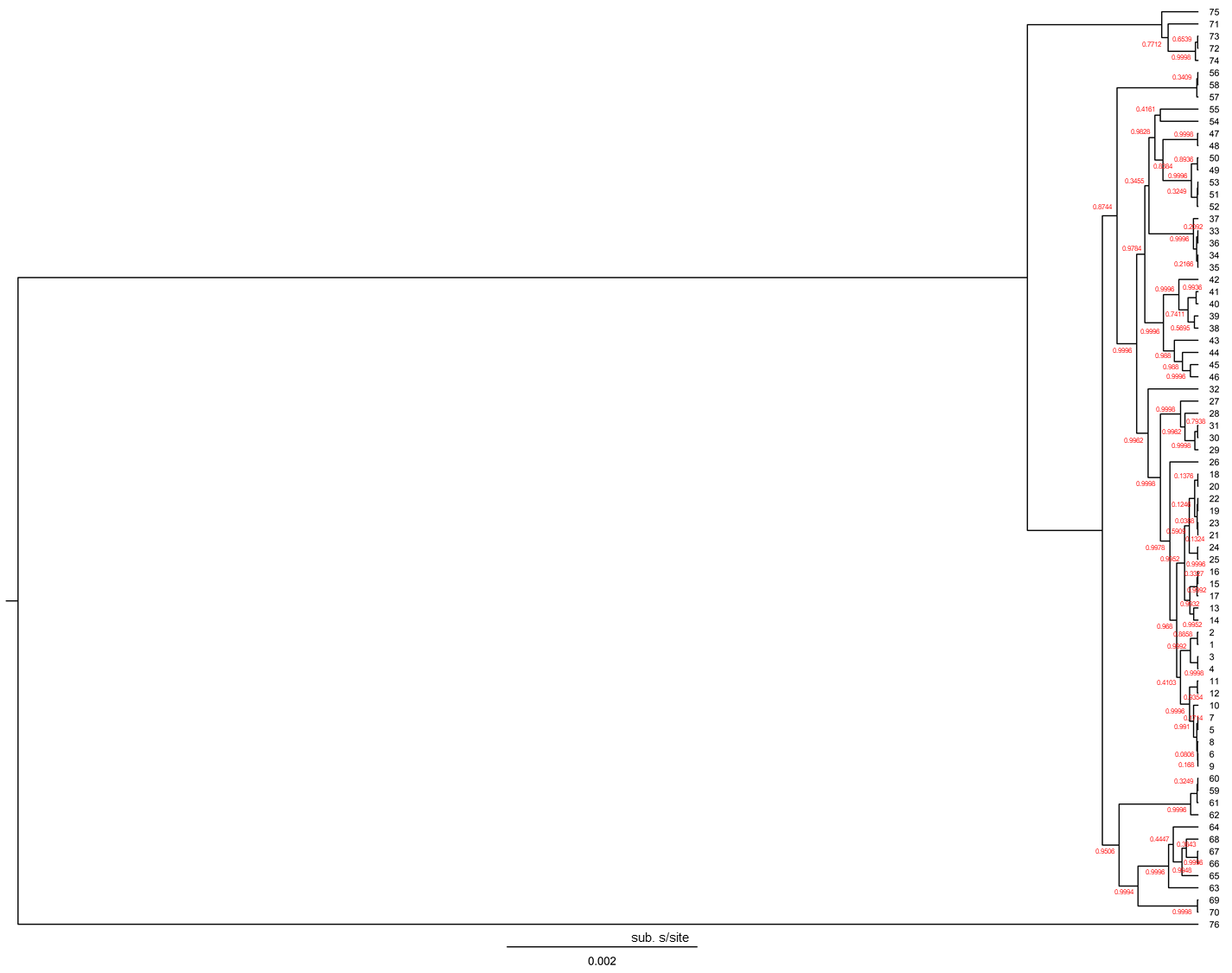


**Supplementary Figure 1.** **Bayesian tree of *Ae. albopictus* mitogenomes.** The posterior probability for the major nodes in the Bayesian tree is reported in red.

**Supplementary Figure 2. Reconstruction of the tiger mosquito dispersal history as depicted by Bayesian phylogeographic diffusion in continuous space.** Line colors were modified according to Figure 2, when a correspondance could be detected. Unlike Figure 2, where also historical data were taken into consideration, arrowsheads (directions of movements) are not an outcome of this analysis. Red shades are proportional to the time of presence of tiger mosquitoes in specific geographic regions, with darker shades of red for older dates.

**Supplementary Figure 3.** Bayesian Skyline Plot (BSP) showing the population size trend of the *Ae. Albopictus*. The Y axis indicates the effective number (Ne) of females. The thick solid line is the median estimate and the blue shading shows the 95% highest posterior density limits. The dotted line highlights the ~120 years ago expansion of haplogroup A1.
